# Associations among individual cultural values, innovation effectiveness, and transformational leadership—evidence from international university students in Türkiye

**DOI:** 10.3389/fpsyg.2026.1786404

**Published:** 2026-08-14

**Authors:** Sümeyye Özburak, Hakan Kitapçı

**Affiliations:** 1 Department of Business Administration, Graduate School of Social Sciences, Gebze Technical University, Kocaeli, Türkiye; 2 Faculty of Arts and Social Sciences, Department of Management Information Systems, Mudanya University, Bursa, Türkiye; 3 Department of Business Administration, Gebze Technical University, Kocaeli, Türkiye

**Keywords:** Hofstede’s cultural dimensions, individual cultural values, innovation effectiveness, international students, transformational leadership

## Abstract

This study examines the associations among individual cultural values, innovation effectiveness, and transformational leadership among international students in higher education institutions in the Marmara Region of Türkiye. Adopting a quantitative, cross-sectional design, the study addresses the culture–leadership–innovation nexus at the individual level. While prior research has predominantly focused on innovation at the organizational level, evidence regarding individual cultural values and leadership processes remains limited, particularly in higher education and multicultural contexts. To address this gap, data were collected from 623 international students with prior internship or work experience, ensuring that leadership perceptions were grounded in real organizational settings. Cultural values were measured using Hofstede’s framework, transformational leadership through the Multifactor Leadership Questionnaire, and innovation effectiveness as a unidimensional construct. The findings indicate that individual cultural values are positively associated with innovation effectiveness. Transformational leadership was positively associated with innovation effectiveness and statistically accounted for part of the observed association between individual cultural values and innovation effectiveness. In addition, a statistically significant but limited interaction was identified, suggesting that the association between individual cultural values and innovation effectiveness varies slightly across different levels of transformational leadership. By examining innovation effectiveness at the individual level and distinguishing between the indirect statistical association and moderating role of transformational leadership, the study provides a more nuanced understanding of the culture–leadership–innovation relationship. Given the cross-sectional and single-source nature of the data, all findings should be interpreted as statistical associations rather than evidence of causal relationships.

## 1 Introduction

Why do individuals exposed to similar organizational environments differ in their ability to generate and implement innovative ideas? Increasing competition, rapid change, and technological advancement have expanded organizational activities beyond local boundaries, creating a highly dynamic and international business environment. In this context, innovation has become a critical factor for achieving sustainable competitive advantage and organizational performance (Agazu and Kero, 2024).

Although innovation has predominantly been examined at the organizational level, the individual-level related factor of innovative behaviour remain insufficiently explored. In particular, the association of individual cultural values with innovation-related behaviours is not yet fully understood. To address this gap, this study focuses on individual-level constructs to avoid cross-level inference bias and to provide a clearer understanding of the culture–leadership–innovation relationship. International university students constitute a particularly relevant context because they interact within culturally diverse educational environments while simultaneously bringing distinct cultural value orientations shaped by their countries of origin.

In this study, cultural values are conceptualized as individual-level constructs reflecting personal value orientations rather than national-level characteristics. Accordingly, Hofstede’s framework is not interpreted at the national level but is used as an analytical tool to capture individual differences in cultural orientations. Existing literature presents mixed findings regarding the role of cultural values in innovation. While some studies suggest that certain cultural orientations foster creativity and innovative behaviour, others indicate that cultural norms may constrain innovation by limiting risk-taking and experimentation. These inconsistencies suggest that examining only the direct relationship between cultural values and innovation may provide an incomplete understanding of the associations between these constructs.

Similarly, although transformational leadership is frequently associated with innovation-related outcomes, its relationship with innovation effectiveness has not been fully clarified in the literature. In particular, empirical findings regarding the nature of the association among transformational leadership, individual cultural values, and innovation effectiveness remain mixed. Some studies have examined transformational leadership as an intervening variable, whereas others have focused on its potential boundary role within these relationships. Taken together, these inconsistencies highlight the need for further research examining the relationships among cultural values, transformational leadership, and innovation effectiveness (Podsakoff et al., 2016). Accordingly, this study adopts a theoretical framework that examines the relationships among individual cultural values, transformational leadership, and innovation effectiveness at the individual level. Within this framework, transformational leadership is examined as both a mediating variable and an exploratory moderating condition. This dual role is theoretically relevant because transformational leadership may be associated with the relationship between cultural values and innovation effectiveness in different ways. In the mediation analysis, transformational leadership is examined as a variable that may account for part of the association between cultural values and innovation effectiveness, whereas in the moderation analysis it is examined as a contextual condition under which this association may vary. Innovation extends beyond technological advancement and reflects a broader process that includes behavioral, organizational, and leadership dimensions (Kreiterling, 2023). Within this process, individual cultural values are associated with how individuals perceive uncertainty, respond to change, and engage in innovative behaviour. Although the relationship betweencultural values and behaviour has been widely examined (Shane, 1993; House, 2004), this relationship remains underexplored at the individual level in the context of innovation.

Hofstede’s Cultural Dimensions Theory provides a useful framework for examining the relationship between cultural values and individual behaviour. These dimensions are associated with individuals’ openness to change, risk-taking tendencies, and responses to uncertainty, all of which have been linked to innovation-oriented behaviour (Hofstede, 1984; Taras et al., 2010; Beugelsdijk et al., 2013).

Furthermore, leadership has been identified as an important factor in studies examining the relationship between cultural values and innovation. Transformational leadership is particularly relevant in this context, as it is associated with a motivating and psychologically supportive environment in which individuals’ engagement in innovative activities has frequently been observed (Bass and Avolio, 1995; Gumusluoglu and Ilsev, 2009; Frazier et al., 2017; Hilton et al., 2023).

Despite the growing body of research, two key gaps remain. First, studies examining the joint effects of individual cultural values and transformational leadership within a unified model are limited. Second, empirical research focusing on international students in higher education contexts remains scarce.

The primary aim of this study is to examine the relationship between individual cultural values and innovation effectiveness and to clarify the role of transformational leadership within this relationship. Specifically, the study investigates the associations among cultural values, transformational leadership, and innovation effectiveness and examines transformational leadership as a potential mediating variable. Given the cross-sectional design, the findings are interpreted as associations rather than causal relationships.

In addition, the potential moderating role of transformational leadership is examined on an exploratory basis. This analysis provides additional insight into whether the relationship between cultural values and innovation effectiveness differs across varying levels of transformational leadership. By focusing on international students with prior internship or work experience in higher education institutions in the Marmara Region of Türkiye, this study contributes to the literature by offering a micro-level perspective on innovation and by providing a clearer distinction between the mediating and moderating roles of transformational leadership.

## 2 Theoretical framework and hypothesis development

This section outlines the conceptual foundations of the key variables and formulates the study hypotheses based on the relationships discussed in the literature.

### 2.1 Individual cultural values

Culture has long been recognized as an important factor related to how individuals perceive their environment, interpret information, and guide their behaviour. According to Hofstede, cultural values represent mental frameworks that shape individuals’ interpretations of events and their behavioural responses within social and organizational contexts (Hofstede, 2001a,b). Hofstede conceptualizes culture through five key dimensions: power distance, individualism–collectivism, uncertainty avoidance, masculinity–femininity, and long-term versus short-term orientation (Minkov and Hofstede, 2011). These dimensions continue to provide a widely used theoretical foundation in contemporary research (Zheng et al., 2025; de Souza Sant’Anna, 2025; Venaik and Brewer, 2010).

Although Hofstede’s framework was originally developed to compare national cultures, subsequent research has shown that cultural values can also be meaningfully examined at the individual level. Individuals within the same national context may exhibit different cultural orientations, highlighting the importance of micro-level analysis (Yoo et al., 2011). Accordingly, this study adopts Hofstede’s framework as a behaviour-oriented lens to examine cultural values at the individual level rather than as a tool for comparing national cultures.

At the individual level, cultural values are closely associated with innovation-related behaviours. For instance, individuals with higher levels of individualism tend to engage in greater risk-taking and exhibit stronger innovative tendencies (Taras et al., 2012). Similarly, the ability to cope with uncertainty has been linked to higher levels of innovative behaviour (Castañeda and Ramírez, 2021). These findings suggest that cultural values are associated with differences in openness to change, risk tolerance, and willingness to engage in innovation.

However, despite the growing body of research, the relationship between individual cultural values and innovation remains theoretically inconsistent. While some studies emphasize the facilitating role of certain cultural orientations, others suggest that cultural norms may constrain innovation by reinforcing conformity and risk aversion. This inconsistency suggest that cultural values alone may not fully account for variations in innovation-related outcomes.

Accordingly, this study adopts a perspective that examines the relationship between individual cultural values and innovation effectiveness at the individual level. Cultural values are associated with how individuals perceive and respond to innovation-related processes. Accordingly, this study extends previous research by examining cultural values, transformational leadership, and innovation effectiveness within a single conceptual framework. Therefore, this study extends previous research by examining cultural values, transformational leadership, and innovation effectiveness within a single conceptual framework.

Despite these inconsistencies, previous research has linked cultural values to key psychological and behavioural tendencies associated with innovation, including openness to new ideas, tolerance for ambiguity, long-term orientation, and willingness to challenge established practices. Collectively, these characteristics have frequently been reported alongside higher levels of innovation effectiveness.

H1: Individual cultural values are positively associated with innovation effectiveness.

### 2.2 Innovation effectiveness

Innovation effectiveness refers to the extent to which innovative activities are associated with intended outcomes. It reflects not only the generation of new ideas but also their successful implementation and the benefits derived from innovation processes (Crossan and Apaydin, 2010). Similarly, Klein and Sorra (1996) define innovation effectiveness in terms of the outcomes achieved through the implementation of innovations, including improvements in efficiency and performance. In this respect, innovation effectiveness is conceptualized as a construct reflecting the successful implementation and practical realization of innovative ideas in the present study.

Although innovation effectiveness is often conceptualized as a multidimensional construct (Calantone et al., 2002; Latif et al., 2017), this study adopts a unidimensional operationalization to capture overall innovation outcomes at the individual level. This approach provides a parsimonious representation and is consistent with prior research focusing on general innovation performance rather than its specific dimensions.

While innovation has traditionally been examined at the organizational level, recent research has increasingly emphasized the role of individuals in innovation processes. In knowledge-intensive environments such as higher education, individuals’ creativity, problem-solving abilities, and innovative behaviours are closely associated with innovation-related outcomes (Anderson et al., 2014).

At the individual level, innovation effectiveness is associated with characteristics such as risk-taking, tolerance for uncertainty, learning orientation, and responsiveness to change (Aldahdouh et al., 2019). Individuals who question existing practices, develop alternative solutions, and make decisions under uncertainty tend to exhibit higher levels of innovation effectiveness (Shane, 1993; Hughes et al., 2018; Anderson et al., 2014).

In addition, innovation effectiveness has been linked to the broader cultural context in which individuals operate. Cultural values are associated with how individuals perceive innovation, evaluate uncertainty, and respond to change (Taras et al., 2010; Gu et al., 2025). Accordingly, a comprehensive understanding of innovation effectiveness requires consideration of individual cultural values.

In line with the theoretical framework, innovation effectiveness is conceptualized as a key study variable reflecting individuals’ ability to generate, develop, and implement innovative ideas in practice.

### 2.3 Transformational leadership and innovation effectiveness

Although transformational leadership is frequently associated withinnovation, the literature does not provide a fully consistent understanding of the relationship between transformational leadership and innovation effectiveness. While many studies report positive associations between transformational leadership and innovation-related outcomes, others suggest that relationships may vary across contextual and individual-level conditions These mixed findings indicate the need for further examination of the patterns linking transformational leadership and innovation effectiveness. In contemporary environments characterized by rapid change and uncertainty, innovation effectiveness reflects not only the generation of new ideas but also individuals’ ability to develop and implement those ideas successfully Within this context, leadership has been widely discussed as a factor associated with innovation-related attitudes and behaviours.

Transformational leadership is particularly relevant because it emphasizes motivation, shared vision, and the cognitive and emotional development of followers (Bass and Avolio, 1996). Previous research has also reported positive associations between transformational leadership and psychological capital, which is related to individuals’ engagement in innovative activities (Njaramba, 2024). Similarly, transformational leadership has been linked to learning orientation, adaptability, and openness to change, all of which are frequently associated with innovation-related outcomes n (Li et al., 2015; Schwarz, 2017).

In addition, prior studies suggest that transformational leadership is associated with creativity and innovative behaviour among followers (Amabile et al., 2004; Oldham and Cummings, 1996; Lee et al., 2020). Individuals who perceive higher levels of support, recognition, and intellectual stimulation from their leaders may report greater willingness to question existing practices and explore alternative approaches.

Furthermore, transformational leadership has been associated with trust and psychological safety within organizational settings, both of which have been linked to the expression and development of new ideas (Hughes et al., 2018). Collectively, these findings suggest a positive relationship between transformational leadership and innovation effectiveness at the individual level.

H2: Transformational leadership is positively associated with innovation effectiveness.

### 2.4 Mediation effect: cultural values-leadership-innovation effectiveness

Existing research has largely examined cultural values and leadership separately in relation to innovation, resulting in a fragmented understanding of how these constructs are associated with one another. In particular, the relationships among cultural values, transformational leadership, and innovation effectiveness have not been sufficiently examined within a single framework. While some studies report a direct relationship between cultural values and innovation effectiveness, others have examined leadership, psychological safety, and knowledge sharing in relation to these variables. However, empirical findings regarding the relationships among these constructs remain limited and inconclusive, highlighting the need for a more comprehensive examination of cultural values, leadership, and innovation effectiveness. In this study, transformational leadership is examined as an intervening statistical construct that may account for part of the observed association between individual cultural values and innovation effectiveness. Cultural values are associated with differences individuals perceive uncertainty, respond to change, and approach risk (Yoo et al., 2011; Zheng et al., 2025).

However, leadership processes have frequently been discussed alongside both cultural values and innovation-related outcomes within organizational contexts.

Previous research suggests that transformational leadership is associated with both individual cultural values and innovation effectiveness, indicating that it may be relevant for understanding the relationship between these constructs. Transformational leadership has been associated with intellectual stimulation, psychological support, and knowledge sharing, all of which have been linked to innovation effectiveness (Lee et al., 2020; Newman et al., 2017; Saif et al., 2024). Accordingly, this study adopts an associational framework in which transformational leadership may statistically account for part of the observed association between cultural values and innovation effectiveness.

Individuals with different cultural orientations may report different perceptions of transformational leadership behaviours.

Consequently, cultural values, transformational leadership, and innovation effectiveness may exhibit statistically related patterns.

H3: Transformational leadership statistically accounts for part of the association between individual cultural values and innovation effectiveness.

#### 2.4.1 Transformational leadership as a potential moderator

The moderating role of leadership in the relationship between cultural values and innovation remains theoretically debated. While leadership is often assumed to strengthen or weaken this relationship, empirical findings are mixed and do not provide a consistent pattern. This suggests that the conditional role of leadership is not yet fully established.

A key explanation for this variability lies in the interaction between individual cultural values and leadership perceptions.

Cultural values are associated with differences in how individuals interpret and respond to leadership behaviours (Dorfman et al., 2012).

Cultural values are associated with differences in individuals’ attitudes toward uncertainty, risk-taking, and change, which are directly related to innovation behaviour. However, the relationship of these values on innovation outcomes is not constant and may depend on the leadership environment (House, 2004; Anderson et al., 2014).

In this context, transformational leadership can be considered a contextual condition under which the association between cultural values and innovation effectiveness may vary.

. Rather than altering individuals’ cultural orientations, transformational leadership may be associated with organizational environments in which existing value tendencies are expressed in different ways.

Accordingly, the relationship between individual cultural values and innovation effectiveness may vary depending on the level of transformational leadership. Under higher levels of transformational leadership, individuals may be more likely to leverage their cultural orientations in ways that support innovation. Therefore, transformational leadership is expected to function as a moderating condition in the relationship between individual cultural values and innovation effectiveness.

H4: The association between individual cultural values and innovation effectiveness varies across levels of transformational leadership.

## 3 Method

### 3.1 Research model

This study examines associations among individual cultural values, transformational leadership, and innovation effectiveness within an integrated conceptual individual cultural values, transformational leadership, and innovation effectiveness within an integrated conceptual framework. Within this framework, individual cultural values, transformational leadership, and innovation effectiveness are examined as theoretically related constructs at the individual level.

Transformational leadership is examined in two complementary ways. First, it is evaluated as a variable that may statistically account for part of the observed association between individual cultural values and innovation effectiveness. Second, it is examined as a contextual factor under which the association between individual cultural values and innovation effectiveness may vary.

The research was designed as a quantitative, cross-sectional study. The proposed hypotheses were examined using regression-based analyses and PROCESS macro models. In the mediation analysis (PROCESS Model 4), individual cultural values (ICV) were specified as the focal independent variable, transformational leadership (TL) as the intervening statistical variable, and innovation effectiveness (IE) as the dependent variable. In the moderation analysis (PROCESS Model 1), ICV was specified as the focal independent variable, IE as the dependent variable, and TL as the moderator.

The analytical framework reflects theoretically derived associations among variables. However, because the data were collected using a cross-sectional and single-source design, the analyses do not establish temporal precedence or causal direction. Accordingly, all findings are interpreted as statistical associations consistent with the proposed theoretical framework rather than as evidence of causal relationships.

**Figure 1:**
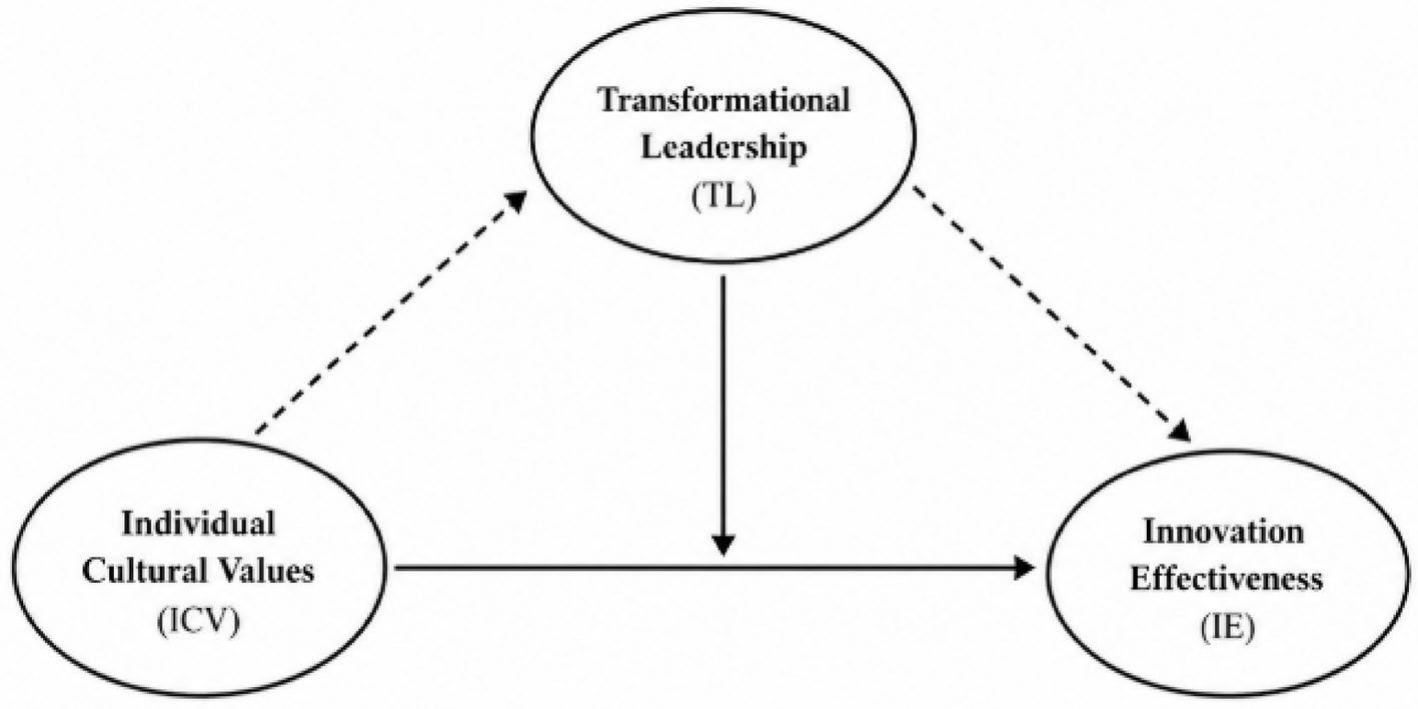
Conceptual association model. The arrows represent theoretically specified statistical associations examined in the study and should not be interpreted as causal pathways.

This study was conducted in accordance with ethical standards and approved by the Ethics Committee of Gebze Technical University (No: E-43633178-199-199365; Date: 15 May 2025). Informed consent was obtained from all participants prior to data collection. Participation was voluntary, and confidentiality and anonymity were ensured throughout the research process (Figure 1).

### 3.2 Research population and sample

The population of this study consists of international students enrolled in higher education institutions in the Marmara Region of Türkiye during the 2024–2025 academic year. The Marmara Region hosts a large number of international students from diverse cultural backgrounds, making it a suitable context for examining culturally driven variables.

The sample comprises 623 international students who have completed an internship or have been involved in organizational activities. To ensure that participants could meaningfully evaluate leadership behaviours, only individuals with prior work or internship experience were included in the study. Participants were asked to evaluate the managers or supervisors they directly interacted with during these experiences, ensuring that perceptions of transformational leadership were grounded in real organizational contexts.

Due to the absence of a clearly defined sampling frame for this specific population, purposive sampling was employed. The sample size was determined based on accessibility and adequacy for statistical analysis. In particular, the sample size exceeds commonly recommended thresholds for regression, mediation, and moderation analyses, thereby providing sufficient statistical power for hypothesis testing. However, the use of purposive sampling may introduce selection bias, and therefore the findings should be interpreted with caution in terms of generalizability. Participation was voluntary, and data were collected through both face-to-face and online surveys.

In studies where a clearly defined sampling frame is not available, non-probability sampling techniques such as purposive sampling are considered appropriate, particularly when the research targets specific subgroups with relevant characteristics (Saunders et al., 2023; Etikan et al., 2016). In the present study, the focus on international students with prior work or internship experience necessitated the use of purposive sampling to ensure that participants could meaningfully evaluate leadership behaviours in real organizational contexts.

Moreover, the adequacy of the sample size was evaluated based on statistical power considerations rather than population representativeness. According to recent methodological guidelines, large sample sizes provide more stable parameter estimates and sufficient statistical power for multivariate analyses (Hair et al., 2019; Kline, 2023). Therefore, while the sampling approach limits generalizability, it is consistent with the study’s analytical objectives and provides a robust basis for examining the proposed relationships at the individual level.

When examining the demographic characteristics of the sample, 44.6% of the participants were female and 55.4% were male. In terms of age distribution, 49.4% were between 18 and 21, 46.7% were between 22 and 25, and 3.9% were between 26 and 29 years old. Regarding work or internship experience, 34.5% had 0–6 months of experience, 54.4% had 6–12 months, and 10.1% had more than 12 months of experience. This distribution indicates that the majority of participants had at least basic exposure to organizational processes.

The sample is characterized by significant national diversity. Participants originate from various regions, including the Middle East (e.g., Lebanon, Yemen, Iran, Iraq, Syria), Africa (e.g., Sudan, Egypt, Ethiopia, Algeria), Central Asia (e.g., Turkmenistan, Afghanistan, Pakistan), and Europe and the Caucasus (e.g., Greece, Bulgaria, Georgia, Azerbaijan). This diversity provides a suitable context for examining individual cultural values. The demographic characteristics of the sample are presented in Table 1.

**Table 1:** Demographic characteristics of participants.

Variable	Category	Frequency (*n*)	Percentage (%)
Gender	Female	278	44.6
	Male	345	55.4
Age group	18–21	308	49.4
	22–25	291	46.7
	26–29	24	3.9
Work/internship experience	0–6 Month	215	34.5
	6–12 Month	345	55.4
	12 Month+	63	10.1
Nationality (regional)	Middle East (Lebanon, Yemen, Iran, Iraq, Syria, etc.)	200	32.1
	Africa (Sudan, Egypt, Ethiopia, Algeria, etc.)	144	23.1
	Central Asia (Turkmenistan, Afghanistan, Pakistan)	78	12.5
	Europe & Caucasus (Greece, Bulgaria, Georgia, Azerbaijan)	132	21.2
	Other	69	11.1
Total		623	100.0

In general, the sample size (*N* = 623) and cultural diversity allow for a multidimensional examination of the relationships between individual cultural values and transformational leadership and innovation effectiveness. As a result, the research provides a robust dataset for examining associations between individuals’ cultural differences and organizational behaviours.

### 3.3 Data collection tool

Data were collected using a structured questionnaire consisting of four sections. All items in the questionnaire were arranged on a 5-point Likert scale (1 = Strongly Disagree, 5 = Strongly Agree). Three scales with proven validity and reliability in the literature were used in preparing the questionnaire.

#### 3.3.1 Individual cultural values scale

The study used Saylık’s (2019) Turkish adaptation of Hofstede’s cultural values scale. Hofstede’s cultural dimensions were originally developed at the national level; however, subsequent research has demonstrated that these dimensions can also be meaningfully operationalized at the individual level as personal cultural orientations (Yoo et al., 2011; Taras et al., 2010, 2016).

In this study, cultural values are examined strictly at the individual level, and no inferences are made regarding national culture. Accordingly, Hofstede’s framework is used as an individual-level analytical tool rather than a representation of national culture. This approach is particularly appropriate for studies involving international student samples, where within-group cultural variability is expected. Accordingly, the focus is on personal value orientations rather than country-level cultural characteristics.

The scale examines cultural values across five dimensions defined by Hofstede: power distance, perceived authority, group relations, risk, and perceived uncertainty. The Cronbach’s Alpha value for the individual cultural values scale was found to be 0.761, indicating an acceptable level of reliability (Nunnally and Bernstein, 1994).

#### 3.3.2 Transformational leadership scale

In this study, transformational leadership was measured using the multidimensional scale developed by Bass and Avolio (1995). The Turkish adaptation of the scale, as presented in the studies by Cemaloğlu (2007) and Şirin and Yetim (2009), was utilized. The scale consists of four dimensions: idealized influence, inspirational motivation, intellectual stimulation, and individualized consideration. The Cronbach’s Alpha value for the scale was found to be 0.920, indicating a high level of reliability (Nunnally and Bernstein, 1994).

#### 3.3.3 Innovation effectiveness scale

In this study, innovation effectiveness was measured using the original scale developed by Calantone et al. (2002) and adapted into Turkish by Vayni (2017). Although innovation effectiveness is frequently conceptualized as a multidimensional construct in the literature, recent studies emphasize that the operationalization of innovation depends on the research objective and level of analysis. When the focus is on overall innovation outcomes rather than specific dimensions, a global and unidimensional measurement approach is considered appropriate (Saunila, 2020; OECD/Eurostat, 2018).

In line with this perspective, prior empirical research has also treated innovation as an overall outcome variable reflecting general innovation performance (Rajapathirana and Hui, 2018). Accordingly, a unidimensional scale was preferred in this study to capture individuals’ overall perception of innovation effectiveness. Furthermore, the exploratory factor analysis results support a single-factor structure, reinforcing the appropriateness of a unidimensional measurement in this study.

The scale includes items related to generating new ideas, developing creative solutions, using knowledge, and adapting to innovation. The Cronbach’s Alpha value for the scale was found to be 0.832, indicating a high level of reliability (Nunnally and Bernstein, 1994).

### 3.4 Data collection process

Data for the study were collected through both face-to-face and online surveys. Participants were invited to participate through university campuses, student communities, and international offices. Prior to data collection, participants were informed about the purpose of the study, and participation was entirely voluntary. Confidentiality and anonymity were ensured throughout the research process.

To reduce potential response bias, participants were instructed to base their responses on their actual experiences in organizational settings. In addition, the questionnaire was designed to minimize common method bias by using clear and concise items and ensuring that responses were provided independently. Data collection was conducted in accordance with ethical research principles.

### 3.5 Common method bias

In this study, data were collected from the same participants using a single questionnaire in a cross-sectional design, which may increase the risk of common method bias (Podsakoff et al., 2003). To mitigate this risk, several procedural remedies were applied. Participants were assured of anonymity and confidentiality and were informed that the data would be used solely for academic purposes. In addition, items were presented in random order, and participants were instructed to base their responses on their actual experiences.

As a statistical check, Harman’s single-factor test was conducted. The results indicated that the first factor accounted for 21.62% of the total variance, which is below the commonly accepted threshold of 50%. This suggests that common method bias is unlikely to be a serious concern. However, given the limitations of Harman’s single-factor test, the results were interpreted with caution, and both procedural and statistical remedies were considered in evaluating the findings.

### 3.6 Data analysis

Data were analysed using SPSS 26. In addition, confirmatory factor analysis (CFA) was conducted using AMOS to assess the construct validity of the measurement model. Model fit was evaluated using multiple indices, including the goodness-of-fit index (GFI), adjusted goodness-of-fit index (AGFI), root mean square residual (RMR), normed fit index (NFI), relative fit index (RFI), parsimony normed fit index (PNFI), and parsimony ratio (PRATIO). The evaluation of model fit was based on commonly accepted threshold values in the literature (Hair et al., 2019; Kline, 2023). The chi-square statistic was not considered as the sole criterion due to its sensitivity to large sample sizes (Kline, 2023).

All variables in this study were conceptualized, measured, and analysed at the individual level. Accordingly, the study does not involve multi-level analysis, and no constructs are interpreted at the national or organizational level. All relationships were examined within a single-level framework to avoid level-of-analysis bias. Individual cultural values represent personal cultural orientations, transformational leadership reflects individuals’ perceptions of their leaders, and innovation effectiveness captures self-reported innovation outcomes.

Prior to the analyses, the distributional properties of the variables were examined. The skewness and kurtosis values of Individual Cultural Values (ICV), Transformational Leadership (TL), and Innovation Effectiveness (IE) ranged between −3 and +3, indicating that the data were normally distributed (Kalaycı, 2010; Tabachnick and Fidell, 2013).

Reliability analyses were conducted using Cronbach’s alpha. The coefficients were 0.761 for ICV, 0.920 for TL, and 0.832 for IE, indicating acceptable to high levels of internal consistency (Büyüköztürk, 2018). The reliability results are presented in Table 2.

**Table 2:** Reliability levels of the scales.

Scale	Number of items (*n*)	Cronbach’s alpha (*α*)
Individual cultural values (ICV)	26	0.761
Transformational leadership (TL)	20	0.920
Innovation effectiveness (IE)	6	0.832

Prior to factor analysis, Kaiser–Meyer–Olkin (KMO) and Bartlett’s test of sphericity were conducted. The KMO values were 0.838 for ICV, 0.939 for TL, and 0.850 for IE. Bartlett’s test results were significant for all scales (*p* < 0.001), indicating the suitability of the data for factor analysis.

Exploratory factor analysis showed that the ICV scale retained a five-factor structure after removing Items 21 and 22 due to cross-loadings, preserving the theoretical structure of Hofstede’s dimensions with minor refinement. The TL scale maintained its original four-factor structure, with loadings ranging from 0.499 to 0.948. For the IE scale, a single-factor structure was obtained, with loadings between 0.502 and 0.837, supporting its use as a unidimensional construct reflecting overall innovation outcomes.

The relationships between variables were examined using Pearson correlation analysis, and hypotheses were tested using regression analysis. Although individual cultural values were measured as a multidimensional construct, a composite index was used in regression analyses to capture their overall effect. This approach is consistent with prior research focusing on general behavioral tendencies rather than dimension-specific effects (Taras et al., 2010).

Multicollinearity diagnostics indicated no concern (VIF = 1.200; Tolerance = 0.833), as values were within acceptable thresholds (Hair et al., 2019).

The proposed mediation model (H3) was tested using Hayes’ PROCESS macro (Version 4.2, Model 4) with 5,000 bootstrap resamples and 95% bias-corrected confidence intervals. The proposed moderation model (H4) was examined using PROCESS Model 1.

In this analysis, ICV was specified as the independent variable, IE as the dependent variable, and TL as the moderator. Independent variables were mean-centred automatically by the PROCESS macro.

In line with best practices, results were reported using unstandardized coefficients (B), standard errors (SE), *t*-values, exact *p*-values, and 95% confidence intervals (BootLLCI and BootULCI).

The proposed moderation model was evaluated based on the significance of the interaction term and the change in explained variance (Δ*R*^2^).

Given the cross-sectional design, mediation findings were interpreted as statistical associations rather than causal relationships (Maxwell and Cole, 2007; Hayes, 2017).

Finally, group differences were examined using ANOVA and Tukey post-hoc tests for age, length of service, and nationality, and independent samples *t*-tests for gender.

## 4 Findings

This section presents the results of descriptive statistics, correlation analyses, regression analyses, and the tests of mediating and moderating effects.

### 4.1 Descriptive statistics

Descriptive statistics for the main variables of the study—Individual Cultural Values (ICV), Transformational Leadership (TL), and Innovation Effectiveness (IE)—are presented in Table 3.

**Table 3:** Descriptive statistics.

Variable	*N*	Min	Max	(Mean)	Std. deviation (SD)	Skewness	Kurtosis
Individual cultural values (ICV)	623	1.69	4.73	3.3702	0.41826	−0.093	0.568
Transformational leadership (TL)	623	1.40	5.00	3.9283	0.61667	−0.670	0.761
Innovation effectiveness (IE)	623	1.00	5.00	3.2515	0.73703	−0.323	0.984

The mean scores indicate that participants reported relatively higher levels of transformational leadership perceptions (M = 3.93) compared with individual cultural values (M = 3.37) and innovation effectiveness (M = 3.25).

The fact that the skewness and kurtosis values fall within the +3 and −3 limits indicates that the variables meet the assumption of normal distribution (Kalaycı, 2010; Tabachnick and Fidell, 2013).

### 4.2 Exploratory and confirmatory factor analysis

To evaluate the construct validity of the measurement instruments, both exploratory factor analysis (EFA) and confirmatory factor analysis (CFA) were conducted. EFA was initially performed to examine the underlying factor structure of the scales, followed by CFA using AMOS to provide additional evidence for the adequacy of the measurement model.

Although all scales had been previously validated, EFA was conducted to examine whether their factor structures were replicated in a multicultural sample of international students. CFA was subsequently performed to assess the adequacy of the measurement model.

#### 4.2.1 Exploratory factor analysis

Exploratory factor analysis (EFA) was conducted to examine the factor structures of the scales used in the study. The results indicate that the Individual Cultural Values (ICV) scale exhibits a five-factor structure, explaining 52.63% of the total variance. The Transformational Leadership (TL) scale also demonstrates a four-factor structure, explaining 58.75% of the total variance. The Innovation Effectiveness (IE) scale exhibits a single-factor structure, explaining 55.30% of the total variance. During the exploratory factor analysis, two items (Item 21 and Item 22) exhibited significant cross-loadings across multiple factors, indicating a lack of discriminant validity. Therefore, these items were removed to achieve a clearer and more interpretable factor structure. Following their removal, a factor structure broadly consistent with the theoretical framework was obtained. All factor loadings were above 0.40, indicating acceptable factor structure and supporting the suitability of the scales for further analysis (Hair et al., 2019). A summary of the factor analysis results is presented in Table 4.

**Table 4:** Factor analysis results.

Variable	Number of factor	KMO	Bartlett (*p*)	Explained total variance (%)	Factor load range
Individual cultural values (ICV)	5	0.840	<0.001	52.63%	0.574–0.828
Transformational leadership (TL)	4	0.939	<0.001	58.753%	0.499–0.948
Innovation effectiveness (IE)	1	0.850	<0.001	55.302%	0.502–0.837

#### 4.2.2 Confirmatory factor analysis

Following the exploratory factor analysis, confirmatory factor analysis (CFA) was conducted using AMOS to provide additional evidence for the validity of the measurement structure. The CFA results indicated that the measurement model demonstrated acceptable overall fit. The goodness-of-fit indices were above commonly recommended thresholds, with GFI = 0.942 and AGFI = 0.936. Similarly, the incremental fit indices supported the adequacy of the model, with NFI = 0.919 and RFI = 0.915. The parsimony-adjusted indices also indicated acceptable model fit, with PNFI = 0.874 and PRATIO = 0.952. In addition, the RMR value (0.075) was within acceptable limits, supporting the adequacy of the model fit.

The standardized factor loadings generally supported the adequacy of the measurement structure. Most item loadings exceeded the recommended threshold of 0.50, particularly for transformational leadership and innovation effectiveness constructs. Although several items demonstrated relatively lower factor loadings, all items were retained in order to preserve the theoretical integrity of the original scales and because the overall model fit indices indicated acceptable model fit (Hair et al., 2019).

The higher-order structure of individual cultural values was also examined to assess whether the dimensions could be represented within a unified measurement structure. The findings generally support the multidimensional nature of cultural values at the individual level, consistent with prior studies emphasizing that cultural orientations may operate heterogeneously across individuals (Taras et al., 2010; Yoo et al., 2011).

**Figure 2:**
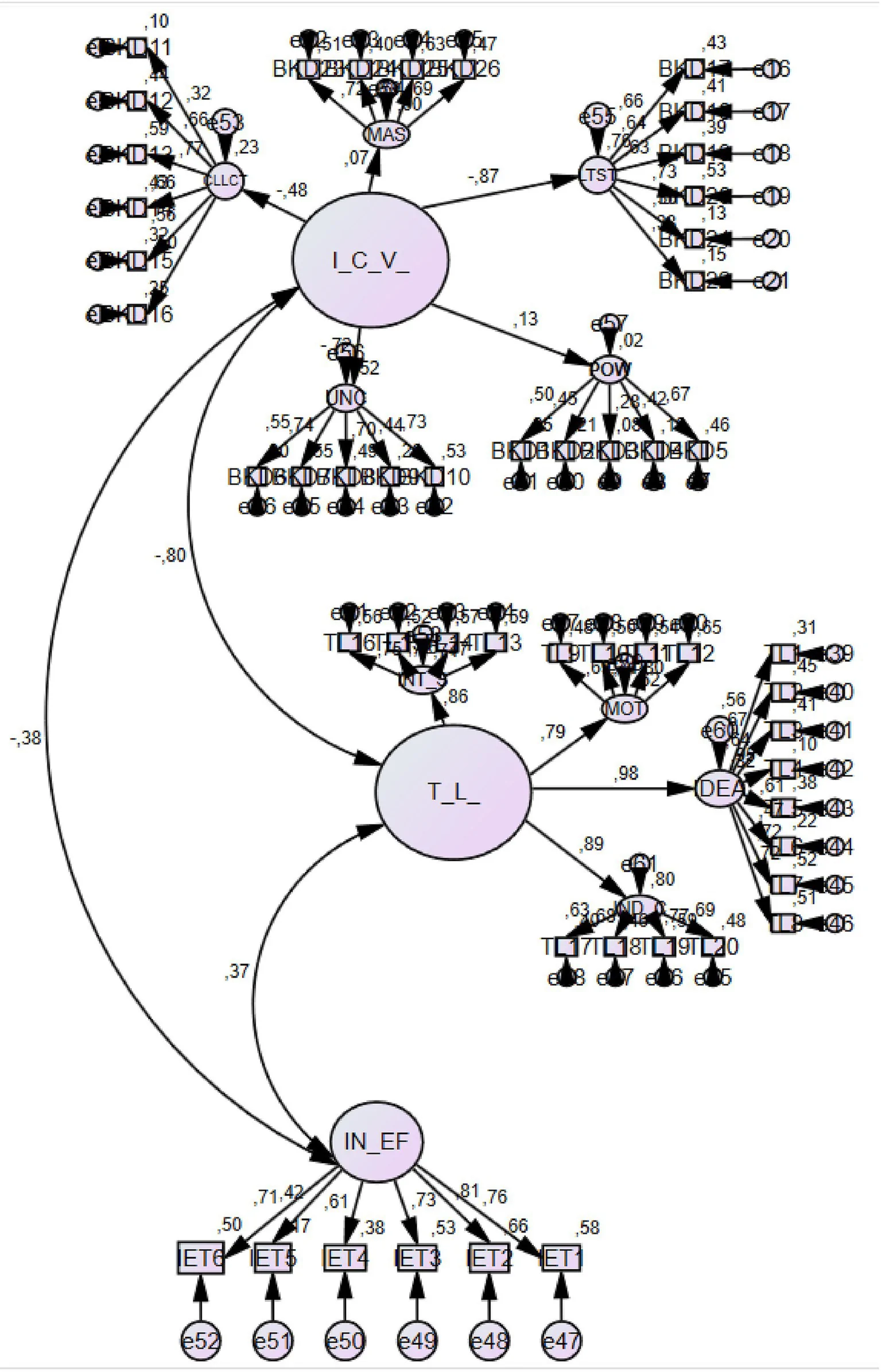
Confirmatory factor analysis (CFA) measurement model with standardized estimates. Standardized estimates are presented on the paths. I_C_V = individual cultural values; T_L_ = transformational leadership; IN_EF = innovation effectiveness.

A CFA measurement model with standardized estimates is presented in Figure 2. The model fit indices are summarized in Table 5, while the standardized factor loadings are presented in Table 6.

**Table 5:** Model fit indices.

Fit index	Value	Threshold	Interpretation
GFI	0.942	≥0.90	Good fit
AGFI	0.936	≥0.90	Good fit
RMR	0.075	≤0.08	Acceptable
NFI	0.919	≥0.90	Good fit
RFI	0.915	≥0.90	Good fit
PNFI	0.874	≥0.50	Acceptable
PRATIO	0.952	≥0.50	Acceptable

**Table 6:** Standardized factor loadings from confirmatory factor analysis.

Construct	Loading range
Power distance	0.280–0.675
Collectivism	0.319–0.771
Uncertainty avoidance	0.444–0.741
Masculinity	0.634–0.793
Long-term orientation	0.357–0.726
Idealized influence	0.320–0.723
Inspirational motivation	0.692–0.804
Intellectual stimulation	0.724–0.765
Individualized consideration	0.635–0.768
Innovation effectiveness	0.417–0.809

Standardized factor loadings below 0.40 were retained to preserve the theoretical structure of the original scales and due to acceptable overall model fit indices.

As shown in Figure 2, the CFA results generally support the adequacy of the measurement structure and provide additional evidence for construct validity.

As shown in Table 5, the CFA results demonstrate that the measurement model achieved an acceptable-to-good overall fit according to commonly recommended thresholds.

The standardized factor loadings generally support the measurement structure. For transformational leadership and innovation effectiveness, most item loadings fall within acceptable ranges, indicating that the observed variables adequately represent their respective latent constructs (Hair et al., 2019). However, relatively low factor loadings were observed for some items, particularly within the individual cultural values scale, suggesting a more complex structure.

The higher-order structure of individual cultural values was also examined to assess whether the five dimensions could be represented within a unified measurement structure. The findings suggest that individual cultural values reflect a multidimensional structure at the individual level, consistent with prior studies emphasizing heterogeneous cultural orientations across individuals (Taras et al., 2010; Yoo et al., 2011).

While several dimensions demonstrated strong standardized loadings, relatively lower loadings were observed for some items within the individual cultural values scale. This pattern is consistent with previous studies reporting greater measurement complexity when Hofstede’s cultural dimensions are operationalized at the individual level in culturally heterogeneous samples. Given the multidimensional nature of the construct and the theoretical importance of preserving all cultural dimensions, these items were retained in the final measurement model.

The standardized factor loadings obtained from the CFA are summarized in Table 6. Overall, the loading ranges indicate acceptable construct representation for the majority of the measurement items.

### 4.3 Correlation analysis

Pearson correlation analysis was conducted to examine the relationships among Individual Cultural Values (ICV), Transformational Leadership (TL), and Innovation Effectiveness (IE). The results are presented in Table 7.

**Table 7:** Correlation analysis between variables.

Variables	Individual cultural values	Transformational leadership	Mean	Standard deviation
Individual cultural values (ICV)			3.37	0.41
Transformational leadership (TL)	0.409**		3.92	0.61
Innovation effectiveness (IE)	0.412**	0.334**	3.25	0.73

**p < 0.001.

The findings indicate that all variables are positively and significantly correlated with each other. A moderate positive relationship was found between ICV and IE (*r* = 0.412, *p* < 0.001). Similarly, ICV is positively correlated with TL (*r* = 0.409, *p* < 0.001). In addition, TL shows a positive and significant relationship with IE (*r* = 0.334, *p* < 0.001), indicating a low-to-moderate association.

All correlation coefficients are below 0.80, indicating no serious multicollinearity concern among the variables.

### 4.4 Regression analysis

#### 4.4.1 Relationship between individual cultural values and innovation effectiveness

The regression results for Model 1 are presented in Table 8. The overall model is statistically significant [*F*(1, 621) = 127.204, *p* < 0.001]. This result indicates that individual cultural values are significantly associated with innovation effectiveness. In practical terms, this finding suggests that individuals with different cultural value orientations tend to differ meaningfully in their capacity to generate and implement innovative ideas, highlighting the importance of cultural factors at the individual level.

**Table 8:** Model 1 regression analysis results.

Variable	*B*	Std. error	*Β*	*T*	*p*
Fixed	0.803	0.219	–	3.669	<0.001
ICV	0.727	0.064	0.412	11.278	<0.001

The results indicate a positive and statistically significant relationship between Individual Cultural Values and Innovation Effectiveness (*β* = 0.412, *p* < 0.001). The model explains 17% of the variance in Innovation Effectiveness (*R*^2^ = 0.170). The results indicate a positive and statistically significant relationship between individual cultural values and innovation effectiveness (*β* = 0.412, *p* < 0.001). This suggests that individuals reporting stronger innovation-supportive cultural value orientations also tended to report higher levels of innovation effectiveness. This finding highlights the importance of cultural value orientations in understanding innovative behaviour at the individual level Furthermore, the model explains 17% of the variance in innovation effectiveness (*R*^2^ = 0.170), highlighting the relevance of individual cultural values in understanding innovation. Accordingly, H1 is supported.

#### 4.4.2 Regression analysis results

The regression results for Model 2 are presented in Table 9. The overall model is statistically significant [*F*(1, 621) = 78.010, *p* < 0.001].

**Table 9:** Regression analysis results.

Variable	*B*	Std. error	*Β*	*T*	*p*
Fixed	1.683	0.180	–	9.363	<0.001
TL	0.399	0.045	0.334	8.832	<0.001

The results indicate a positive and statistically significant relationship between transformational leadership and innovation effectiveness (*β* = 0.334, *p* < 0.001). This suggests that leaders who exhibit transformational behaviours—such as articulating a clear vision, inspiring individuals, and providing individualized support—are associated with environments in which the generation and implementation of innovative ideas are more frequently reported.”

Furthermore, the model explains 12% of the variance in innovation effectiveness (*R*^2^ = 0.120), indicating a meaningful association between transformational leadership and innovation effectiveness. Accordingly, H2 is supported. Accordingly, H2 is supported.

#### 4.4.3 Examination of the proposed mediation model

Mediation analysis was conducted using Hayes’ PROCESS macro (Model 4) with 5,000 bootstrap samples and 95% confidence intervals (Hayes, 2017).

In the first step, a positive and statistically significant relationship was observed between Individual Cultural Values (ICV) and Transformational Leadership (TL) (*B* = 0.6024, *t* = 11.1563, *p* < 0.001). The model accounted for 16.7% of the variance in TL (*R*^2^ = 0.167), indicating that the variables were meaningfully associated. This suggests that individuals with stronger cultural value orientations are more likely to perceive or exhibit higher levels of transformational leadership.

In the second step, when both ICV and TL were included in the model predicting Innovation Effectiveness (IE), both variables had significant positive effects. ICV remained positively and significantly associated with innovation effectiveness (*B* = 0.5835, *t* = 8.4284, *p* < 0.001), and TL was also positively and significantly associated with innovation effectiveness (*B* = 0.2376, *t* = 5.0599, *p* < 0.001).

The model explained 20.3% of the variance in IE (*R*^2^ = 0.203), indicating a modest but meaningful level of explanatory power. These findings suggest that both cultural values and leadership behaviours are significantly associated with differences in individuals’ innovation effectiveness.

Bootstrapping results indicated a statistically significant indirect association (*B* = 0.1431, BootSE = 0.0326). The 95% confidence interval did not include zero (BootLLCI = 0.0833, BootULCI = 0.2100), supporting the proposed mediation model. These results are consistent with the proposed mediation model involving Transformational Leadership and suggest an indirect association among the variables.

The indirect association estimated through PROCESS was statistically significant. However, because the data are cross-sectional and obtained from a single source, this finding should not be interpreted as definitive evidence regarding the temporal ordering of the variables. Rather, it indicates a pattern of associations consistent with the proposed theoretical model. Accordingly, H3 is supported (Table 10).

**Table 10:** Indirect association analysis results.

Path	*B*	SE	*T*	*p*	LLCI	ULCI
ICV → TL	0.6024	0.0540	11.1563	<0.001	0.4964	0.7085
TL → IE	0.2376	0.0470	5.0599	<0.001	0.1454	0.3298
ICV → IE (Direct)	0.5835	0.0692	8.4284	<0.001	0.4475	0.7194
Indirect association (ICV → TL → IE)	0.1431	0.0326	–	–	0.0833	0.2100

#### 4.4.4 Examination of the proposed moderation model

The findings indicated a statistically significant interaction between individual cultural values and transformational leadership (*B* = 0.1915, SE = 0.0856, *t* = 2.2360, *p* = 0.0257). Furthermore, the 95% confidence interval did not include zero (LLCI = 0.0233, ULCI = 0.3597).

In addition, the inclusion of the interaction term led to a statistically significant but very small increase in the explained variance of innovation effectiveness (Δ*R*^2^ = 0.0064, *p* = 0.0257). This indicates that, although the interaction term reached statistical significance, its practical contribution to the model is limited.

These findings suggest that the relationship between individual cultural values and innovation effectiveness varies slightly across different levels of transformational leadership. However, the magnitude of the interaction was relatively small, indicating limited variation across levels of transformational leadership. Therefore, while H4 received statistical support, the observed interaction was relatively small and should be interpreted with caution. Accordingly, the findings related to the proposed moderation model should be viewed as exploratory and warrant further investigation in future research (Table 11).

**Table 11:** Moderating role of transformational leadership.

Predictor	B	SE	*T*	*p*	LLCI	ULCI
ICV	−0.1653	0.3419	−0.4834	0.629	−0.8366	0.5061
TL	−0.3804	0.2803	−1.3571	0.175	−0.9308	0.1701
ICV × TL	**0.1915**	0.0856	2.2360	0.0257	0.0233	0.3597

Bold values indicate statistically significant results at *p* < 0.05.

### 4.5 Differences analysis according to demographic values (ANOVA and *t*-tests)

In this part of the study, we examined whether there were significant differences in our research variables, namely individual cultural values (ICV), transformational leadership (TL), and innovation effectiveness (IE) levels, according to the demographic characteristics of the participants. A one-way ANOVA was performed for the age and work experience variables, and an independent samples *t*-test was performed for the gender variable.

#### 4.5.1 Analysis of differences by age group

A one-way ANOVA test was performed to determine the existence of a significant difference in participants’ individual cultural values (ICV), Transformational Leadership (TL), and Innovation Effectiveness (IE) levels according to age groups. Levene’s test results indicated that variances were homogeneous (ICV: *p* = 0.371; TL: *p* = 0.439; IE: *p* = 0.514). There were no significant differences between age groups on the variables [ICV: *F*(2,620) = 0.413, *p* = 0.662]; TL: [*F*(2,620) = 0.599, *p* = 0.54]; IE: [*F*(2,620) = 0.329, *p* = 0.720]. The Türkiye post-hoc analysis results also showed that the mean differences between groups were not significant. Our results indicate that no statistically significant differences were observed across age groups for individual cultural values, innovation effectiveness, and transformational leadership. Therefore, age does not appear to be a differentiating factor within this specific sample. However, this finding should not be generalized beyond the current sample.

#### 4.5.2 Analysis of differences based on length of employment

A one-way ANOVA test was performed to determine whether there were significant differences in individual cultural values (ICV), Transformational Leadership (TL), and Innovation Effectiveness (IE) levels among groups formed based on participants’ length of employment. Levene’s test results showed that the variances were homogeneous (ICV: *p* = 0.636; TL: *p* = 0.155; INE: *p* = 0.335). There was no significant difference between the groups formed according to length of service in terms of individual cultural values and innovation effectiveness variables [ICV: *F*(2,620) = 0.977, *p* = 0.377; IE: *F*(2,620) = 1.002, *p* = 0.368]. However, transformational leadership values differed significantly based on length of service [*F*(2,620) = 3.470, *p* = 0.032]. Tukey post-hoc analysis results indicate that participants with 12 months or more of organizational experience have significantly higher perceptions of transformational leadership than participants with 0–6 months and 6–12 months of organizational experience (*p* = 0.045). More positive evaluations of leadership behaviours were observed among individuals with greater organizational experience.

#### 4.5.3 Gender-based difference analyses

An independent samples *t*-test was conducted to investigate whether there were significant differences between participants’ individual cultural values, transformational leadership, and innovation effectiveness based on their gender. The results of the analyses showed that the variables did not exhibit significant differences based on the gender variable [ICV (*t*(621) = −0.971, *p* = 0.332), TL (*t*(621) = 0.137, *p* = 0.891) and IE (*t*(621) = 0.137, *p* = 0.891)].

## 5 Discussion

This study examined the associations among individual cultural values, Transformational leadership and innovation effectiveness among international students in Türkiye. The findings provide evidence of statistically significant relationships among these constructs and contribute to the growing literature on culture, leadership, and innovation at the individual level.

The findings indicate a positive association between individual cultural values and innovation effectiveness. This result is consistent with previous studies suggesting that cultural orientations are related to differences in openness to change, attitudes toward uncertainty, and innovation-related behaviours (Taras et al., 2010; Castañeda and Ramírez, 2021). Rather than implying that cultural values directly is associated with innovation outcomes, the findings suggest that individuals exhibiting different cultural orientations also tend to report different levels of innovation effectiveness.

The results also indicate a positive association between transformational leadership and innovation effectiveness. This finding is consistent with prior research reporting positive relationships between transformational leadership perceptions and innovation-related outcomes (Bass and Avolio, 1995; Gumusluoglu and Ilsev, 2009). Individuals reporting higher levels of transformational leadership also tended to report higher levels of innovation effectiveness. These findings are broadly consistent with theoretical perspectives emphasizing the relevance of supportive leadership environments in innovation-related contexts.

The mediation analysis revealed a statistically significant indirect association involving transformational leadership. However, it is important to emphasize that this finding should not be interpreted as evidence that transformational leadership causally transmits the effects of cultural values to innovation effectiveness. Given the cross-sectional and single-source nature of the data, the mediation results merely indicate a pattern of statistical associations that is consistent with the proposed theoretical framework. Alternative explanations and causal orderings remain plausible and cannot be ruled out.

The moderation analysis indicated a statistically significant but relatively small interaction association (Δ*R*^2^ = 0.0064). This findings suggests that the association between individual cultural values and innovation effectiveness differs slightly across levels of transformational leadership. Nevertheless, the practical magnitude of this interaction was limited. Therefore, transformational leadership should be viewed primarily as a contextual factor associated with variations in the relationship rather than as a strong boundary condition.

Taken together, the findings suggest that cultural values, leadership perceptions, and innovation effectiveness are meaningfully interconnected at the individual level. However, the observed relationships should not be interpreted as evidence of directional causal processes. Instead, they should be understood as statistically significant associations that may inform future theory development and empirical investigation.

An additional contribution of the study concerns its focus on individual-level cultural values. While Hofstede’s framework has often been applied at the national level, the present study conceptualizes cultural values as individual orientations. This perspective helps avoid cross-level inference problems and provides additional insight into how cultural differences may be associated with innovation-related perceptions and behaviours within multicultural environments.

The demographic analyses indicated relatively limited differences across participant groups. This finding suggests that cultural values and leadership perceptions may be more strongly associated with innovation effectiveness than the demographic characteristics examined in this study. Nevertheless, future research should further investigate these relationships using more diverse samples and research designs.

Overall, the findings contribute to the literature by documenting statistically significant associations among cultural values, transformational leadership, and innovation effectiveness. Future longitudinal, experimental, and multi-source studies are needed to determine whether the observed associations reflect underlying causal processes.

Finally, the cross-sectional design of the study limits the ability to draw causal inferences. Therefore, the findings should be interpreted as associative rather than causal relationships (Maxwell and Cole, 2007; Hayes, 2017). Future research using longitudinal, experimental, and multi-source designs would provide deeper insights into the dynamics of these relationships.

## 6 Conclusion

This study examined the associations among individual cultural values, transformational leadership, and innovation effectiveness among international students in Türkiye. By bringing these constructs together within a single analytical framework, the study contributes to existing research on culture, leadership, and innovation at the individual level.

The findings indicate that individual cultural values are positively associated with innovation effectiveness and that transformational leadership is also positively associated with innovation effectiveness. In addition, transformational leadership was significantly associated with both cultural values and innovation effectiveness. Furthermore, a small but statistically significant interaction was observed between individual cultural values and transformational leadership.

The findings further suggest that cultural values should be considered as multidimensional individual-level orientations rather than as homogeneous national characteristics. This perspective provides a useful framework for understanding cultural diversity within international and multicultural environments.

A notable contribution of the study lies in distinguishing between indirect statistical associations and interaction effects involving transformational leadership. The results indicate that transformational leadership is related to both the association between cultural values and innovation effectiveness and the conditions under which that association may vary.

At the same time, the findings should be interpreted cautiously. Because the study relies on cross-sectional and single-source data, no causal conclusions can be drawn regarding the relationships among the variables. The observed patterns represent statistical associations consistent with the theoretical framework rather than definitive evidence of causal processes.

From a practical perspective, the findings suggest that cultural values, leadership perceptions, and innovation effectiveness are interconnected constructs within multicultural environments. Future research employing longitudinal, experimental, and multi-source designs would be valuable for examining the robustness and potential causal nature of these relationships.

## 7 Limitations and future research

Despite its theoretical and practical contributions, this study has several limitations that should be considered when interpreting the findings.

First, the sample consists exclusively of international students enrolled in higher education institutions in the Marmara Region of Türkiye, which may limit the generalizability of the findings to other populations and contexts. However, it is important to note that all participants had prior internship or work experience and evaluated real supervisors in organizational settings. Therefore, the findings are based on actual leadership experiences rather than hypothetical assessments.

Second, the absence of a clearly defined sampling frame for the target population required the use of purposive sampling, which may introduce selection bias. The sample size was determined based on accessibility and statistical adequacy rather than probabilistic representativeness. Nevertheless, the relatively large sample size (N = 623) exceeds commonly recommended thresholds for regression, mediation, and moderation analyses, thereby providing sufficient statistical power for hypothesis testing. Despite this, the findings should be interpreted with caution in terms of generalizability.

Third, the cross-sectional design of the study restricts the ability to draw causal inferences and to capture dynamic changes in the relationships among variables over time, particularly with regard to the proposed mediation model. Therefore, the mediation findings should be interpreted as statistical associations consistent with the theoretical model rather than definitive evidence of causal processes.

Fourth, the use of self-report measures may introduce common method bias and social desirability effects. Although procedural and statistical remedies were applied to minimize this risk, future studies could provide additional evidence regarding validity by incorporating multi-source data, such as supervisor evaluations or objective performance indicators.

Another limitation concerns the measurement structure of individual cultural values. Although CFA was conducted to assess construct validity, some dimensions of the higher-order cultural values construct exhibited relatively lower factor loadings than others. This indicates that cultural dimensions may not operate as a fully unified construct at the individual level, but rather reflect a multidimensional structure (Taras et al., 2010; Yoo et al., 2011). Future studies may examine each cultural dimension separately or test alternative measurement models using longitudinal or multi-source data.

Finally, the study focuses solely on transformational leadership, which may limit the scope of leadership-related interpretations. Future research could extend the model by examining alternative leadership styles, such as ethical, servant, or inclusive leadership, to provide a more comprehensive understanding of how different leadership approaches interact with individual cultural values in shaping innovation effectiveness. In addition, although a statistically significant interaction was observed, its relatively small magnitude suggests that future research should further examine the relationship between leadership and innovation effectiveness using alternative research designs and samples.

## Data Availability

The original contributions presented in the study are included in the article. Further inquiries can be directed to the corresponding author.
